# Adhesion of Staphylococcus aureus to Corneocytes from Atopic Dermatitis Patients Is Controlled by Natural Moisturizing Factor Levels

**DOI:** 10.1128/mBio.01184-18

**Published:** 2018-08-14

**Authors:** Cécile Feuillie, Pauline Vitry, Maeve A. McAleer, Sanja Kezic, Alan D. Irvine, Joan A. Geoghegan, Yves F. Dufrêne

**Affiliations:** a Institute of Life Sciences, Université Catholique de Louvain, Louvain-la-Neuve, Belgium; b National Children’s Research Centre, Our Lady’s Children’s Hospital, Dublin, Ireland; c Pediatric Dermatology, Our Lady’s Children’s Hospital, Dublin, Ireland; d Clinical Medicine, Trinity College Dublin, Dublin, Ireland; e Coronel Institute of Occupational Health, Academic Medical Center, Amsterdam, The Netherlands; f Department of Microbiology, Moyne Institute of Preventive Medicine, School of Genetics and Microbiology, Trinity College Dublin, Dublin, Ireland; g Walloon Excellence in Life Sciences and Biotechnology (WELBIO), Wavre, Belgium; University of California, Berkeley; University of Washington

**Keywords:** adhesion, atopic dermatitis, biofilms, skin, *Staphylococcus *aureus**

## Abstract

The bacterial pathogen Staphylococcus aureus plays an important role in atopic dermatitis (AD), a chronic disorder that mostly affects children. Colonization of the skin of AD patients by S. aureus exacerbates the disease, but the molecular determinants of the bacterium-skin adhesive interactions are poorly understood. Specifically, reduced levels of natural moisturizing factor (NMF) in the stratum corneum have been shown to be associated with more severe AD symptoms, but whether this is directly related to S. aureus adhesion is still an open question. Here, we demonstrate a novel relationship between NMF expression in AD skin and strength of bacterial adhesion. Low-NMF corneocytes, unlike high-NMF ones, are covered by a dense layer of nanoscale villus protrusions. S. aureus bacteria isolated from AD skin bind much more strongly to corneocytes when the NMF level is reduced. Strong binding forces originate from a specific interaction between the bacterial adhesion clumping factor B (ClfB) and skin ligands. Remarkably, mechanical tension dramatically strengthens ClfB-mediated adhesion, as observed with catch bonds, demonstrating that physical stress plays a role in promoting colonization of AD skin by S. aureus. Collectively, our findings demonstrate that patient NMF levels regulate the strength of S. aureus-corneocyte adhesion, the first step in skin colonization, and suggest that the ClfB binding mechanism could represent a potential target for new therapeutic treatments.

## IMPORTANCE

Bacterium-skin interactions play important roles in skin disorders, yet their molecular details are poorly understood. In this study, we decipher the molecular forces at play during adhesion of Staphylococcus aureus to skin corneocytes in the clinically important context of atopic dermatitis (AD), also known as eczema. We identify a unique relationship between the level of natural moisturizing factor (NMF) in the skin and the strength of bacterium-corneocyte adhesion. Bacterial adhesion is primarily mediated by the surface protein clumping factor B (ClfB) and is enhanced by physical stress, highlighting the role of protein mechanobiology in skin colonization. Similar to a catch bond behavior, this mechanism represents a promising target for the development of novel antistaphylococcal agents.

## INTRODUCTION

Atopic eczema, also known as atopic dermatitis (AD), is a chronic skin disorder that initially starts in childhood, where it affects 20 to 30% of the population in urbanized and developed settings. There is a lower, but still significant prevalence of 5 to 10% in adults (1, 2). Colonization of the skin by Staphylococcus aureus appears to amplify the severity of the disease (3, 4). More severe AD is associated with low natural moisturizing factors (NMF), either due to a primary genetic loss-of-function (LOF) mutation in *FLG* (5) or due to the secondary effects of systemic Th2 inflammation on filaggrin and NMF expression (6, 7). Patients with AD who have an *FLG* LOF mutation are more likely to be heavily colonized by S. aureus (8). Previous work has shown that NMF products inhibit bacterial proliferation (9). The mechanisms through which *FLG* mutations and/or low NMF facilitate or enhance adhesion and colonization behavior of S. aureus are not known. Clarification of this issue may give us new clues to develop therapeutics to reduce skin colonization and infection.

**FIG 1:**
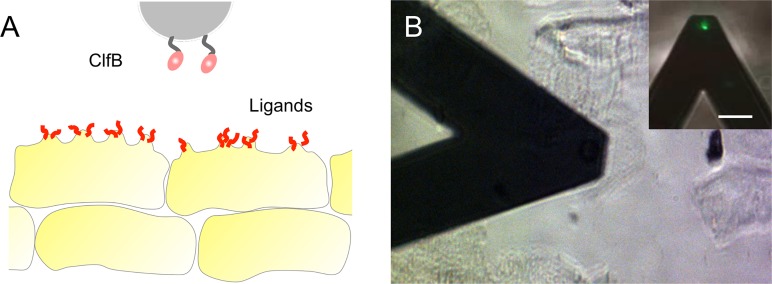
Exploring the forces between Staphylococcus aureus and AD corneocytes. (A) We used AFM-based single-cell force spectroscopy and multiparametric imaging to study the forces between bacterial cells and skin samples from AD patients immobilized on tape strips. Single bacterial probes were prepared by attaching S. aureus bacteria onto AFM cantilevers. (B) Optical microscopy image showing the bacterial AFM probe scanning across the surfaces of corneocytes. (Inset) Fluorescence image of the probe showing that the bacterial cell is alive (BacLight viability kit) (bar, 20 µm).

We recently showed that the bacterial cell surface protein clumping factor B (ClfB) mediates the adhesion of S. aureus to corneocytes from AD patients (10). ClfB binds to the cornified envelope proteins loricrin and cytokeratin (11–13) via the dock, lock, and latch (DLL) mechanism. An important yet unsolved question is whether S. aureus adhesion to AD skin depends on skin NMF levels. In this study, we examine the impact of patient NMF levels on the strength of adhesion between S. aureus and corneocytes, that is, cells found on the outermost surface of the epidermis, the stratum corneum. To study bacterial adhesion in a clinical context, we probe the interaction forces of an AD skin isolate of S. aureus using atomic force microscopy (AFM) (14, 15) (Fig. 1). We investigate corneocytes from patients with two different filaggrin genotypes: “WT” (wild type) where the *FLG* gene is intact, filaggrin is produced in normal quantities, and NMF levels are high, and “HET” where filaggrin is produced at approximately 50% of normal levels due to heterozygous LOF mutations and NMF levels are low (5, 6, 16, 17). We demonstrate a unique relationship between the NMF level and the strength of bacterial adhesion. S. aureus-corneocyte adhesion is primarily mediated by ClfB and dramatically increased when the NMF level is reduced. Strong adhesion forces originate from DLL binding of ClfB to corneocyte ligands and are activated by mechanical force. These results demonstrate that NMF levels in AD skin control the strength of S. aureus adhesion, which may explain why this factor is important in determining the severity of the AD disease. This study highlights the value of AFM to enhance our understanding of the molecular basis of skin diseases. By helping to define the bacterial and host factors contributing to bacterial adhesion to skin, this emerging nanotechnology offers promise for identifying new agents to reduce—or prevent—skin colonization by S. aureus.

## RESULTS

### Surface nanomorphology of AD corneocytes.

**FIG 2:**
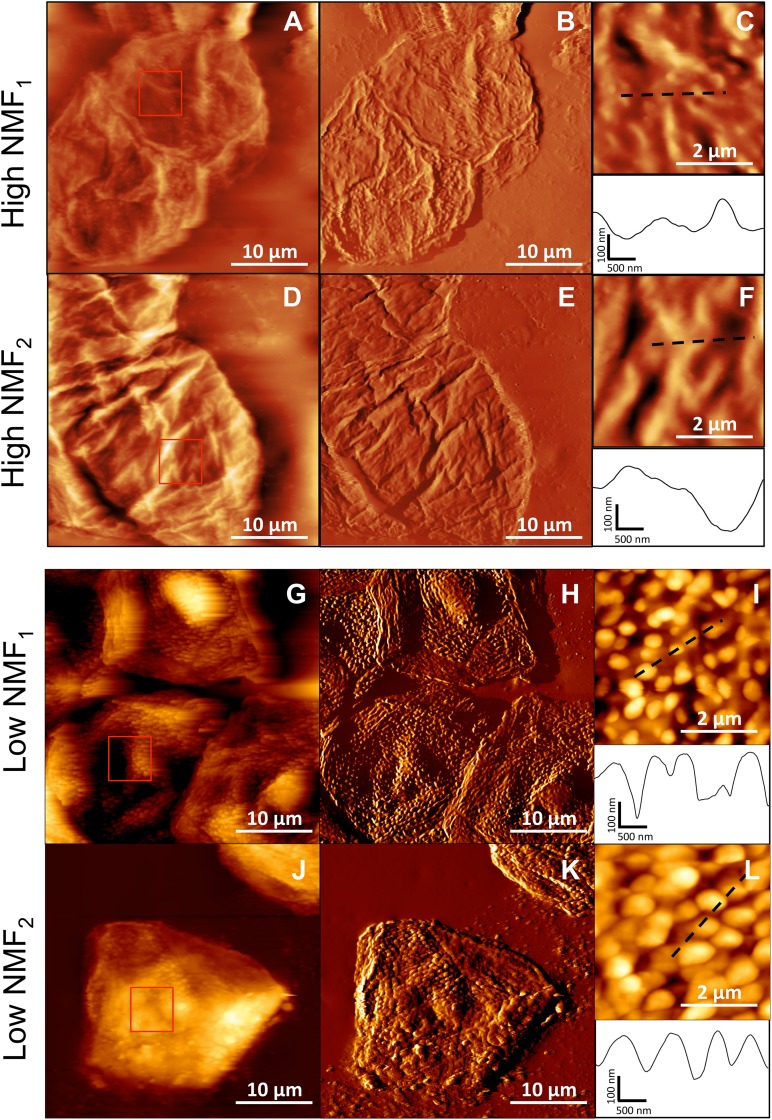
Topographic imaging reveals major structural differences between corneocytes with high and low NFM levels. Height images (z-range = 2 µm) (A, D, G, and J) and corresponding deflection images (B, E, H, and K) recorded in PBS for corneocytes from patients with high NMF levels (high NMF_1_ and high NMF_2_) or low NMF levels (low NMF_1_ and low NMF_2_). (C, F, I, and L) High-resolution height images recorded in the square areas shown in panels A, D, G, and J, together with vertical cross sections taken along the dashed lines.

Throughout this work, we studied skin samples from a total of four patients, two with a WT filaggrin genotype, hereafter designated “high NMF_1_” (NMF level of 0.73 mmol/g of total protein), and “high NMF_2_” (0.74 mmol/g of total protein), and two with a HET genotype, low NMF_1_ (0.14 mmol/g of total protein) and low NMF_2_ (0.17 mmol/g of total protein). As filaggrin deficiency is associated with corneocyte morphological defects, we first imaged the nanoscale surface topography of WT and HET skin samples in buffer solution (Fig. 2). While the structure of high-NMF corneocytes was rather smooth and devoid of any peculiar features (Fig. 2A to F), low-NMF corneocytes showed large numbers of villus protrusions (VPs), about 200 nm in height (Fig. 2G to L). Consistent with earlier reports (10, 18), these data confirm that low NMF levels, due to relative deficiency of filaggrin, lead to altered surface morphology of the corneocytes, and this is associated with high VP numbers. As the number of VPs correlates closely with the level of NMF, we know that patients with these abnormalities will have an association with more severe disease and a characteristic profile with higher pH, greater barrier dysfunction, and a less hydrated stratum corneum. While the profile of low-NMF corneocytes and these morphological abnormalities has been reported in several studies, the mechanisms underlying these projections are unclear, as are the direct structural and functional consequences.

### ClfB-mediated adhesion is tightly controlled by NMF levels.

**FIG 3:**
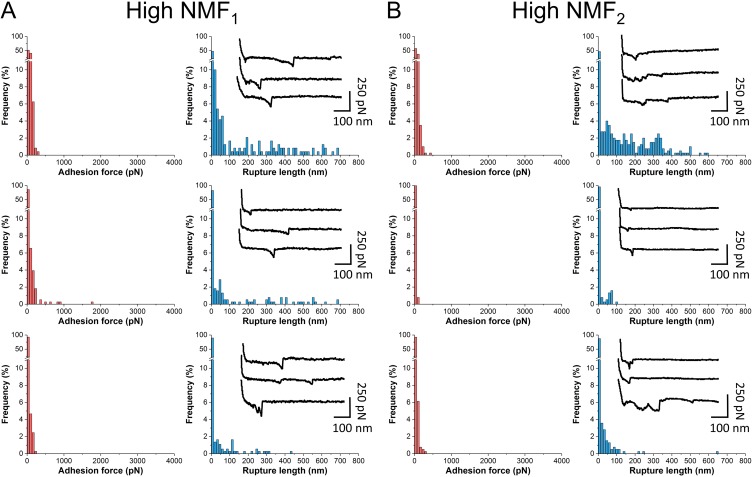
S. aureus AD08 bacteria bind weakly to high-NMF corneocytes. (A and B) Adhesion force and rupture distance histograms with representative force profiles obtained in PBS between S. aureus AD08 bacteria and corneocytes from two patients with high NMF levels. For each patient, three representative bacterium-corneocyte pairs out of a total of seven pairs are shown. See Fig. S1 in the supplemental material for additional bacterium-corneocyte pairs, and see Fig. 5 for a statistical analysis of all samples analyzed.

To capture the interaction forces between S. aureus and corneocytes, single bacterial cells were immobilized onto AFM cantilevers, and force-distance curves were recorded between the bacterial probes and skin samples immobilized on tape strips. We analyzed corneocytes from the upper part of the stratum corneum (second strip). In Fig. 3, we present the results from force measurements between S. aureus AD08 bacteria and corneocytes from two different patients with high NMF levels (for each patient, three representative bacterium-corneocyte pairs are shown; for more pairs, see Fig. S1 in the supplemental material). While some curves featured weak adhesion events with forces of ~50 to 250 pN and rupture lengths generally shorter than ~250 nm, the remaining curves showed no adhesion. The measured forces were not substantially different from one cell pair to another or when comparing the two patients. Hence, high NMF levels are associated with weak bacterial adhesion forces, suggesting that NMF compounds contribute to prevent colonization. This fits well with recent clinical studies showing heavier S. aureus colonization in patients with *FLG* mutations (8).

10.1128/mBio.01184-18.1 FIG S1  Single-cell force spectroscopy of the interaction between S. aureus AD08 and skin of patients with AD. Adhesion force histograms obtained in PBS between additional S. aureus AD08 bacteria and corneocytes from AD patients with high NMF levels (A and B) or low NMF levels (C and D) are shown. For each patient, four additional pairs are presented. Download FIG S1, PDF file, 0.3 MB. Copyright © 2018 Feuillie et al. 2018 Feuillie et al. This content is distributed under the terms of the Creative Commons Attribution 4.0 International license.

**FIG 4:**
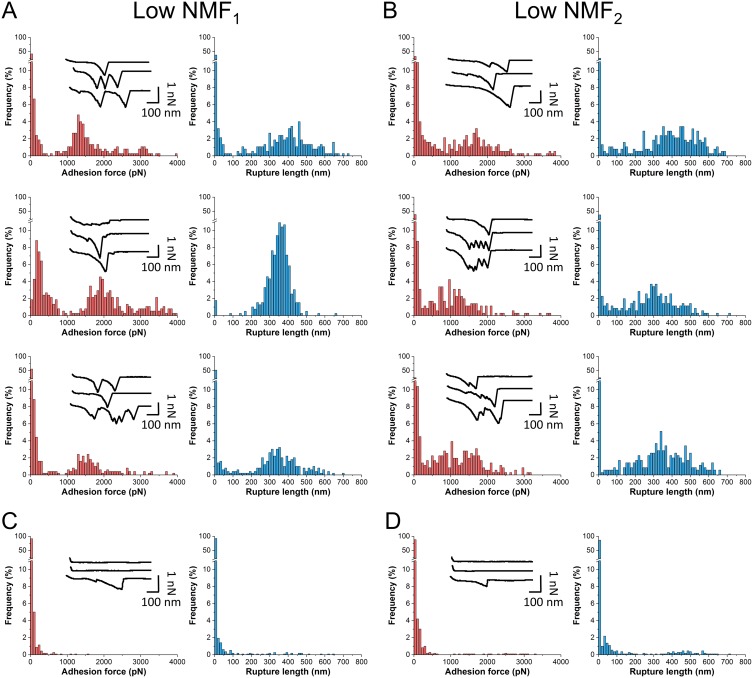
Bacterial adhesion to low-NMF corneocytes is strong and mediated by ClfB. (A and B) Adhesion force and rupture distance histograms with representative force profiles obtained in PBS between S. aureus AD08 bacteria and corneocytes from two patients with low NMF levels. For each patient, three representative bacterium-corneocyte pairs out of a total of seven pairs are shown. (C and D) Data obtained under the same conditions for bacterial cells from the AD08 Δ*clfB* strain. Results obtained for three representative bacterium-corneocyte pairs out of a total of nine pairs are pooled. See Fig. S1 and S2 for additional bacterium-corneocyte pairs, and see Fig. 5 for a statistical analysis of all samples analyzed.

**FIG 5:**
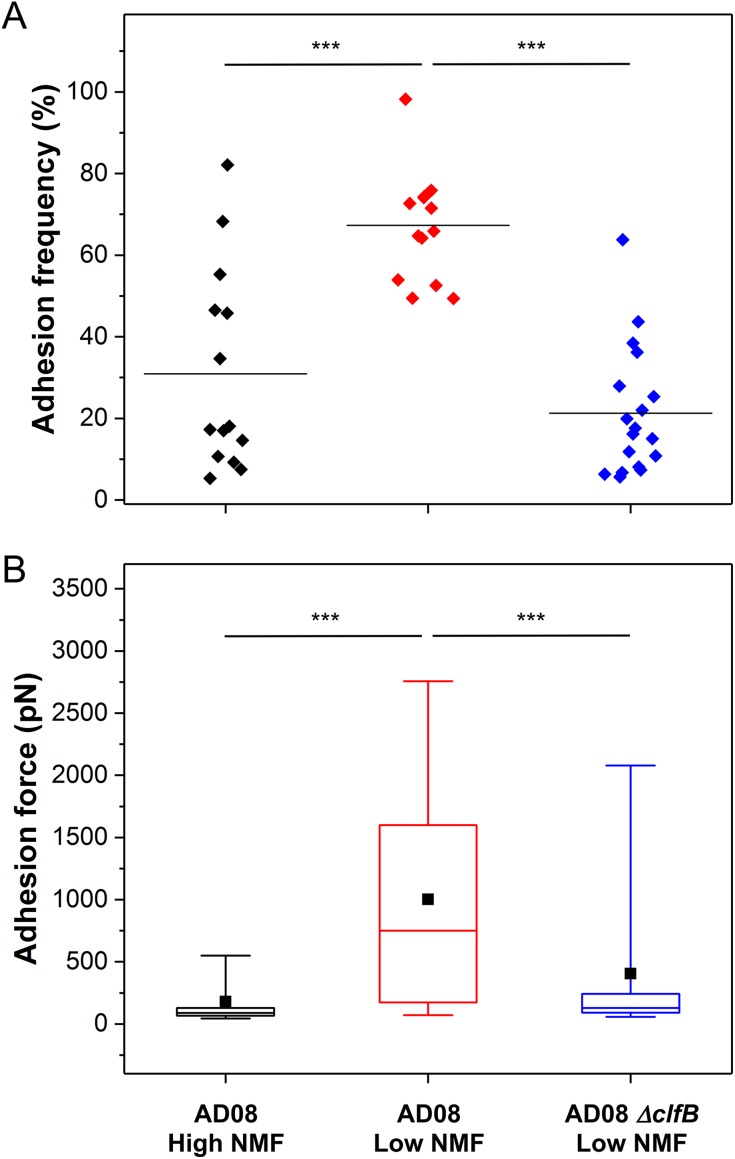
Statistical analysis of the interaction strength between S. aureus and AD corneocytes. (A and B) Adhesion frequencies (A) and adhesion forces (B) recorded between S. aureus AD08 bacteria and corneocytes from AD patients with high NMF levels (two patients; *n* = 1,431 adhesive curves from 14 different cells) or low NMF levels (two patients; *n* = 3,577 curves from 14 cells). Data obtained between low-NMF corneocytes and the AD08 Δ*clfB* strain are also shown (two patients; *n* = 1423 adhesive curves from 18 different cells). The horizontal lines in panel A represent the mean adhesion frequency. Box-charts in panel B show the mean adhesion (full square), median, first and third quartiles (boxes), and range of data without outliers (whiskers, 5 to 95 percentiles). Statistical analysis was performed by one-way ANOVA and Bonferroni *post hoc* tests with a *P* value of <0.001 indicated by three asterisks.

Notably, much stronger forces were observed between S. aureus AD08 bacteria and low-NMF corneocytes (Fig. 4; for more pairs, see Fig. S1). Force curves displayed two types of adhesion events, i.e., weak (or moderate) forces in the ~50 to 500 pN range and strong forces of ~1,500 pN (1,551 ± 163 pN and 1,462 ± 72 pN for the two patients; means ± standard deviations [SDs] for three independent pairs). Such strong forces are in the range of forces expected for a high-affinity DLL binding mechanism (11, 12, 19, 20). As they are close to the forces measured between the S. aureus adhesin ClfB and the squamous epithelial cell envelope protein loricrin (13), we postulated that they may originate from the binding of ClfB to target ligands. To test this idea, we analyzed mutant cells lacking ClfB (AD08 Δ*clfB*). As shown in Fig. 4C and D (for more pairs, see Fig. S2), most strong forces were missing, suggesting that ClfB plays an important role in this interaction. Figure 5 presents a statistical analysis of data obtained on multiple cells from independent cultures, revealing that both the adhesion frequency and adhesion forces were significantly higher on low-NMF corneocytes than on high-NMF corneocytes and that these values were strongly reduced using the AD08 Δ*clfB* strain. All together, these observations show that NMF levels and bacterial adhesion properties are highly correlated, thus indicating that NMF expression is an important factor that governs colonization of AD skin by S. aureus.

10.1128/mBio.01184-18.2 FIG S2  Single-cell force spectroscopy of the interaction between S. aureus AD08 Δ*clfB* and skin of patients with AD. Adhesion force histograms obtained in PBS between additional S. aureus AD08 Δ*clfB* bacteria and corneocytes from AD patients with low NMF levels are shown. For each patient, six additional pairs are presented. Download FIG S2, PDF file, 0.2 MB. Copyright © 2018 Feuillie et al. 2018 Feuillie et al. This content is distributed under the terms of the Creative Commons Attribution 4.0 International license.

To further support that the strong interaction between S. aureus AD08 and corneocytes is associated with AD, we analyzed two control (CTRL) samples, i.e., corneocytes taken from the volar forearm skin of children unaffected by AD and with either no detected *FLG* mutation (hereafter CTRL_WT_; NMF level of 1.32 mmol/g of total protein) or carrying an *FLG* mutation (CTRL_HET_; NMF level of 0.28 mmol/g of total protein). Figure S3 shows nanoscale topographic images of the two skin samples in buffer solution. The surface of both corneocytes was quite smooth and the two corneocytes were devoid of VPs, in contrast with the low-NMF AD corneocytes (Fig. 2G to L). These results indicate that high VP densities are associated with AD symptoms. In Fig. S4, it can be seen that force curves between S. aureus AD08 bacteria and CTRL samples showed adhesive forces of ~500 pN (603 ± 821 pN and 454 ± 444 pN for CTRL_WT_ and CTRL_HET_; means ± SDs for five independent cell pairs). Hence, strong DLL forces of ~1,500 pN were essentially never observed on control samples, showing that ClfB-mediated DLL binding occurs only on skin of patients with AD symptoms.

10.1128/mBio.01184-18.3 FIG S3  Topographic imaging of non-AD skin. Height images (z-range = 1.5 µm) (A and D) and corresponding deflection images (B and E) obtained for corneocytes from children unaffected by AD and presenting either no detected *FLG* mutation (CTRL_WT_; NMF level = 1.32 mmol/g of total protein) or an *FLG* mutation (CTRL_HET_; NMF level = 0.28 mmol/g of total protein). (C and F) High-resolution height images recorded in the square areas shown in panels A and D. Download FIG S3, PDF file, 0.2 MB. Copyright © 2018 Feuillie et al. 2018 Feuillie et al. This content is distributed under the terms of the Creative Commons Attribution 4.0 International license.

10.1128/mBio.01184-18.4 FIG S4  Single-cell force spectroscopy of the interaction between S. aureus AD08 and non-AD skin. (A and B) Adhesion force and rupture distance histograms with representative force profiles obtained in PBS between S. aureus AD08 bacteria and corneocytes from children unaffected by AD, CTRL_WT_ (A) and CTRL_HET_ (B). Download FIG S4, PDF file, 0.5 MB. Copyright © 2018 Feuillie et al. 2018 Feuillie et al. This content is distributed under the terms of the Creative Commons Attribution 4.0 International license.

### Physical stress potentiates bacterium-corneocyte adhesion.

**FIG 6:**
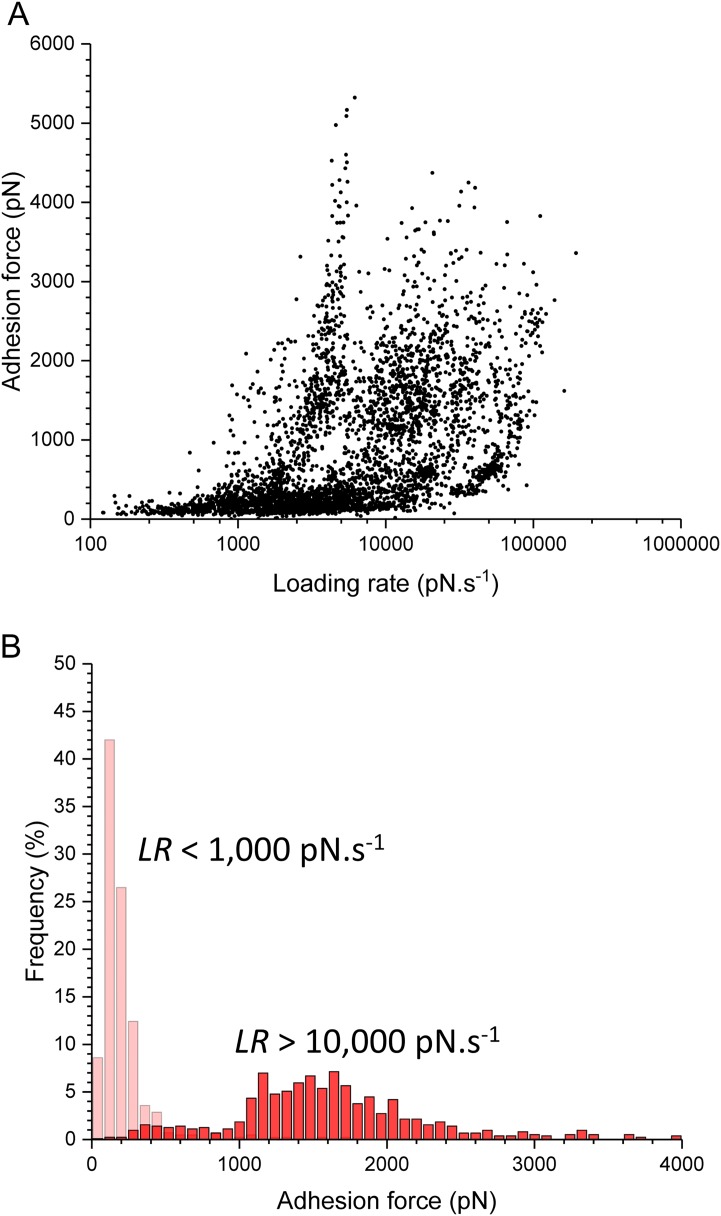
Mechanical tension potentiates bacterium-corneocyte adhesion in low-NMF conditions. (A) Adhesion forces measured at increasing loading rates (LRs) between S. aureus AD08 bacteria and low-NMF_1_ corneocytes (data pooled from 4,009 adhesive peaks on three cell pairs). (B) The force distribution switches with the rate at which force is applied, i.e., while weak bonds dominate at low LR (<1,000 pN ⋅ s^−1^), strong bonds are favored at s high LR (>10,000 pN ⋅ s^−1^). These results support a catch bond model where ClfB-mediated adhesion is enhanced through force-induced conformational changes.

During colonization of the stratum corneum, S. aureus is subjected to numerous physical stresses, such as hydrodynamic flow, cell surface contact, and epithelial turnover (21). We therefore wondered whether the S. aureus-skin interaction could depend on mechanical force. To test this hypothesis, we studied the variation of adhesion forces (*F*) as a function of the rate at which force is applied (loading rate [LR]), and we analyzed the force distribution over discrete ranges of LRs (Fig. 6). At low LRs, only weak forces centered at 146 ± 132 pN were observed, whereas at high LRs, mostly strong forces of 1,520 ± 809 pN were seen, demonstrating that the probability of forming strong bonds dramatically increases with tensile force. This unusual behavior implies that the mechanical stability of ClfB bonds is strongly enhanced by mechanical stress. Flow experiments showed that S. aureus adhesion can be strengthened by high shear forces (22–27), and our data support a model whereby ClfB binds skin ligands via a catch bond-like interaction (28), enabling S. aureus to resist high physical stresses during colonization. Supporting this view, we recently showed that the DLL interaction between ClfB and purified loricrin is strengthened by tensile force (13). Single-molecule experiments suggested that under an external force, the conformation of ClfB changes from a folded, weakly binding state to an extended, strongly binding state. Our finding that physical stress potentiates ClfB-mediated adhesion to corneocytes emphasizes that the mechanics of staphylococcal adhesions play an important role in controlling the adhesion of bacteria to skin.

### ClfB ligands are largely exposed on low-NMF corneocytes.

**FIG 7:**
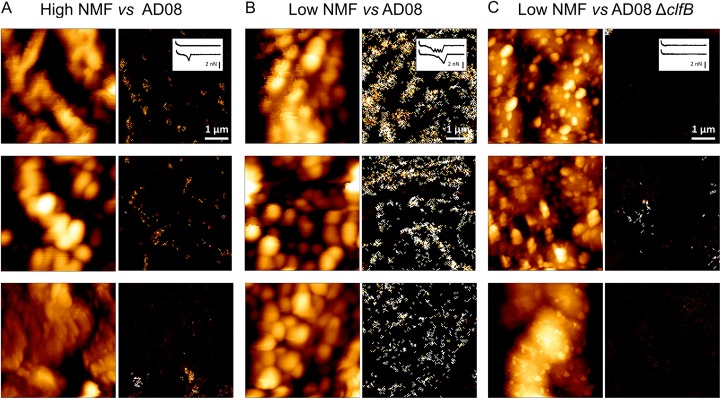
Nanoscale adhesion imaging shows that ClfB ligands are largely exposed on low-NMF corneocytes. (A and B) Simultaneous height (left) and adhesion (right) images of corneocytes recorded in PBS between different S. aureus AD08 cell probes and different high-NMF_2_ (A) or low-NMF_1_ (B) corneocytes. (C) Images obtained on different low-NMF_1_ corneocytes with different AD08 Δ*clfB* cell probes. The insets in the top right images show representative force curves. More images are presented in Fig. S5.

An interesting question is how do adhesive interactions distribute across the corneocyte surface? To answer this, we used a novel high-speed multiparametric imaging technique (29, 30), in which a bacterial probe is used to spatially map bacterium-host adhesive interactions on corneocytes. Figure 7 presents correlated images of the structure and adhesion of low-NMF corneocytes recorded with AD08 bacterial probes (see Fig. S5 for more images from independent samples). Topographic images clearly featured VP structures, although the resolution was limited by the large size of the probe (~1 µm). Interestingly, adhesion images revealed large binding forces of 3,671 pN ± 663 pN (*n* = 727 curves from three independent samples) that were heterogeneously distributed across the corneocyte surface (see the bright pixels in the maps). We note that the ClfB adhesion forces measured here are much higher than those measured above (Fig. 4), reflecting the influence of the loading rate on the strength of adhesion. These observations support the notion that S. aureus adhesion is stronger when VPs are present and explain why low-NMF corneocytes support more S. aureus adhesion than high-NMF corneocytes devoid of VPs.

10.1128/mBio.01184-18.5 FIG S5  Nanoscale adhesion imaging of AD skin using bacterial probes. (A and B) Additional height (left) and adhesion (right) images of corneocytes recorded in PBS between S. aureus AD08 cell probes and high-NMF_2_ (A) or low-NMF_1_ (B) skin samples. (C) Images obtained on low-NMF_1_ corneocytes with AD08 Δ*clfB* cell probes. Download FIG S5, PDF file, 0.2 MB. Copyright © 2018 Feuillie et al. 2018 Feuillie et al. This content is distributed under the terms of the Creative Commons Attribution 4.0 International license.

## DISCUSSION

Bacterium-skin adhesion plays an important role in skin disorders, yet the molecular interactions involved have long been inaccessible to study. We have shown that AFM is a powerful platform to study the forces at play during colonization of the skin by S. aureus in the clinically important context of AD skin disease. By quantifying the forces between S. aureus clinical isolates and corneocytes from AD patients, we have identified a novel relationship between skin NMF levels in AD and the strength of bacterium-corneocyte adhesion, and we found that this interaction is activated by physical stress. Reduced filaggrin, and therefore NMF, expression has been shown to facilitate S. aureus colonization *in vitro* (31) and *in vivo* (8). Our single-molecule experiments elegantly show the molecular mechanism behind these observations.

Our main discovery is that NMF levels determine the strength of ClfB-mediated adhesion, that is, bacterial adhesion is weak at high NMF levels, but very strong when NMF levels are reduced. Strong adhesion originates from mechanically stable DLL bonds between ClfB and skin ligands. While they show rather classical affinity values (19), DLL interactions represent the strongest receptor-ligand bonds ever measured (32). This points to the important notion that, under conditions of high physiological shear forces as seen during skin colonization, measuring bond strengths under tensile loading (out of equilibrium) might be more relevant than measuring affinity values (at equilibrium). The weak adhesion to low-NMF corneocytes exhibited by ClfB-deficient mutants could be mediated by other S. aureus proteins such as SdrC, SdrD, and SasG that have been shown previously to promote adhesion to desquamated nasal epithelial cells (33).

We postulate that the strong ClfB-dependent adhesion originates from an increased exposure or expression of ligand proteins on the stratum corneum surface for the following reasons. First, it is established that filaggrin deficiency is associated with skin defects and exposure of skin proteins (18). Second, topographic images show that the surface morphology of corneocytes from low-NMF skin samples is altered, with a high density of VPs. Third, our multiparametric images demonstrate that strong ClfB bonds are heterogeneously distributed across the corneocyte surface and concentrate mainly on the top of VPs, suggesting that these regions are enriched in ClfB ligands (18).

Another salient feature of our work is that bacterium-skin adhesion is dramatically enhanced by physical stress. That the mechanical stability of ClfB bonds is strongly enhanced by tensile force supports a catch bond mechanism (28) and highlights the role of mechanobiology (34) in regulating the binding activity of S. aureus adhesins. This finding is of biological significance because during adhesion and colonization of the skin, S. aureus is subjected to physical stresses, such as hydrodynamic flow, cell surface contact, and epithelial turnover (16, 34). We thus propose that S. aureus has evolved such a unusual force-enhanced adhesion mechanism to provide the bacteria with a competitive advantage to colonize AD skins under shear stress. This interaction represents a promising target for therapy, and it could be used for the development of new inhibitors to prevent staphylococcal adhesion. In particular, our results show that ClfB is a key mediator of interactions between S. aureus and AD skin, specifically, and suggest that ClfB is a suitable target for new antiadhesion therapies. Inhibition of S. aureus binding to corneocytes through disruption of the interaction between ClfB and its target ligands might reduce skin colonization in AD.

## MATERIALS AND METHODS

### Bacterial strains and growth conditions.

S. aureus wild-type strain AD08, a clinical isolate from a patient with atopic dermatitis (AD) and its isogenic ClfB-deficient mutant, AD08 Δ*clfB* (10), were cultured in tryptic soy broth (TSB) overnight at 37°C under agitation. Before experiments, cells were washed once in prewarmed TSB, and then 100 µl of this suspension was inoculated in 10 ml of fresh prewarmed TSB. Cells were grown at 37°C, under agitation, until an optical density at 600 nm (OD_600_) of 0.3 was reached. For atomic force microscopy (AFM) experiments, cells were harvested by centrifugation, washed twice in phosphate-buffered saline (PBS), and diluted 1:10 in PBS.

### Collection of corneocytes.

Corneocytes were collected by tape stripping on the volar forearm skin of volunteer children with AD and healthy children. All patients were treatment naive (they had not been exposed to topical steroids or antibiotics prior to recruitment). Circular adhesive tape strips (3.8 cm^2^) (D-Squame; Monaderm, Monaco, France) were attached to the skin and pressed for 10 s before being removed. Eight consecutive tape strips containing corneocytes were sampled from the same site on the skin. Each tape strip was placed in a closed vial and stored at −80°C until analysis. For AFM and natural moisturizing factor (NMF) measurements, the second and fourth tape strips were used, respectively. The study was approved by the Research Ethics Committee of Our Lady’s Children’s Hospital Crumlin. All subjects gave written and informed consent. Subjects with AD were recruited from dermatology clinics; unaffected control subjects were recruited when attending the hospital for routine operative procedures.

### NMF determination.

The NMF concentrations have been determined by the high-performance liquid chromatography (HPLC) method described in detail by Dapic et al. (35). Briefly, the NMF components histidine (His), 2-pyrrolidone-5-carboxylic acid (PCA), and urocanic acid (UCA) (*trans* and *cis* isomers) were extracted from each tape strip by adding 500 µl of 25% (wt/wt) ammonia solution. After 2 h of continuous shaking (IKA Vibrax model 2200; IKA Works Inc., Wilmington, NC, USA), the extracts were evaporated to dryness at 60°C (Eppendorf concentrator 5301; Eppendorf AG, Hamburg, Germany), and the residue was dissolved in 500 µl Millipore water and analyzed by HPLC. To compensate for the variable amount of stratum corneum (SC) harvested by a tape strip, total proteins were measured on each tape by a Pierce Micro BCA protein assay kit (Thermo Fisher Scientific, Rockford, IL, USA), and the levels of NMF were expressed as millimoles per gram of total protein.

### Single-cell force spectroscopy.

Bacterial cell probes were obtained by first preparing colloidal probes: a single silica microsphere (6.1-µm diameter; Bangs Laboratories) was attached with a thin layer of UV-curable glue (NOA 63; Norland Edmund Optics) on triangular tip-less cantilevers (NP-O10; Bruker) using a Nanowizard III AFM (JPK Instruments, Berlin, Germany). Colloidal probe cantilevers were then immersed for 1 h in Tris-buffered saline (TBS) (50 mM Tris, 150 mM NaCl [pH 8.5]) containing 4 mg/ml of dopamine hydrochloride (Sigma-Aldrich), rinsed in TBS, and used directly for cell probe preparation. The nominal spring constant of the colloidal probe cantilever was of ~0.06 N/m, as determined by the thermal noise method. Then, 50 µl of a diluted cell suspension was deposited into the petri dish containing corneocytes at a distinct location within the petri dish; 2 ml of PBS was added to the petri dish. The colloidal probe was brought into contact with an isolated bacterium and retracted to attach the bacterial cell; proper attachment of the cell on the colloidal probe was checked using optical microscopy. Cell probes were used to measure bacterium-corneocyte interaction forces at room temperature, using an applied force of 0.25 nN, a constant approach-retraction speed of 1.0 µm/s, and a contact time of 100 ms. Experiments were also conducted while varying the retraction speed, from 100 nm/s to 5 µm/s. Data were analyzed using the data processing software from JPK Instruments (Berlin, Germany). Adhesion force and distance rupture histograms were obtained by calculating the maximum adhesion force and the rupture distance of the last peak for each curve.

To assess statistically significant differences between adhesion force data sets, we performed one-way analysis of variance (ANOVA) and Bonferroni *post hoc* multiple-comparison tests using the 2017 Origin software. Force values obtained for both low-NMF_1_ (7 cells; 1,980 adhesive force curves) and low-NMF_2_ (7 cells; 1,597 adhesive force curves) patients were pooled and considered one data set for the interaction of *S*. *aureus* AD08 bacteria on low-NMF skin. Similarly, we pooled force values obtained for high-NMF_1_ (7 cells; 870 adhesive force curves) and high-NMF_2_ (7 cells; 561 adhesive force curves) patients on one hand, and force values obtained with AD08 Δ*clfB* bacteria on low-NMF_1_ (9 cells; 726 adhesive force curves) and low-NMF_2_ (9 cells; 697 adhesive force curves) patients, on the other hand. A *P* value of <0.001 was considered statistically significant.

### Multiparametric imaging.

Multiparametric images were acquired in PBS using the Quantitative Imaging mode available on the Nanowizard III AFM (JPK Instruments, Berlin, Germany), following the experimental protocol described by Formosa-Dague et al. [30]). Briefly, images were obtained using a S. aureus cell probe, at 128 pixels × 128 pixels, with an applied force kept at 1.0 nN, and a constant approach/retract speed of 40.0 µm/s (z-range of 1 µm). The cantilever’s spring constants were determined by the thermal noise method.
