# Supplementary material for: Adhesion of Staphylococcus aureus to Corneocytes from Atopic Dermatitis Patients Is Controlled by Natural Moisturizing Factor Levels
Source: mBio. 2018 Aug 14;9(4):e01184-18. doi: 10.1128/mBio.01184-18 (PMC6094479; doi:10.1128/mBio.01184-18)
Supplement: FIG S1 [file mbo004184009sf1.pdf]

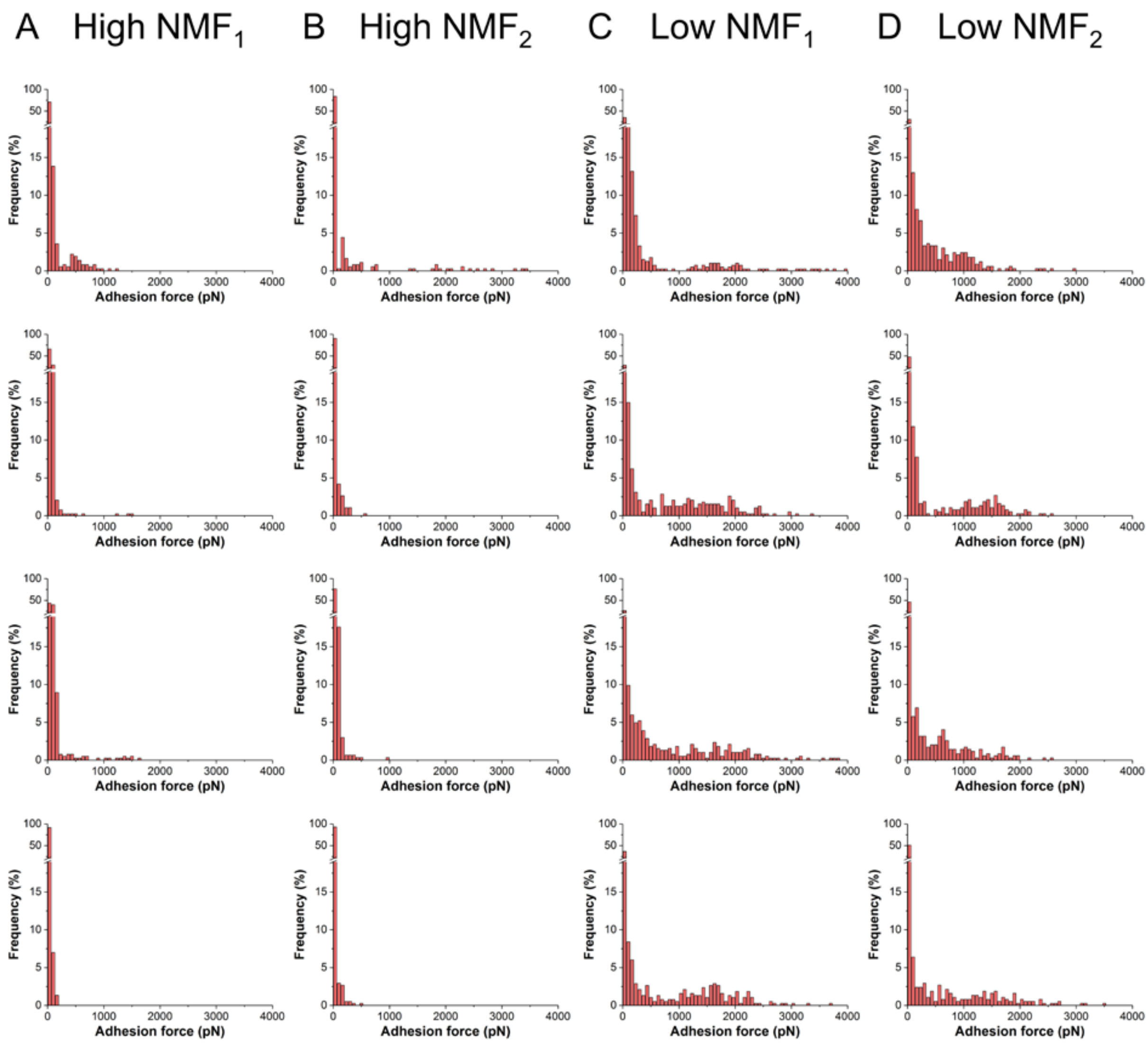

Figure S1. Single-cell force spectroscopy of the interaction between *S. aureus* AD08 and AD skins. Adhesion force histograms obtained in PBS between additional *S. aureus* AD08 bacteria and corneocytes from AD patients with high NMF levels (*A*, *B*) or low NMF levels (*C*, *D*). For each patient, 4 additional pairs are presented.
