# Supplementary material for: Adhesion of Staphylococcus aureus to Corneocytes from Atopic Dermatitis Patients Is Controlled by Natural Moisturizing Factor Levels
Source: mBio. 2018 Aug 14;9(4):e01184-18. doi: 10.1128/mBio.01184-18 (PMC6094479; doi:10.1128/mBio.01184-18)
Supplement: FIG S2 [file mbo004184009sf2.pdf]

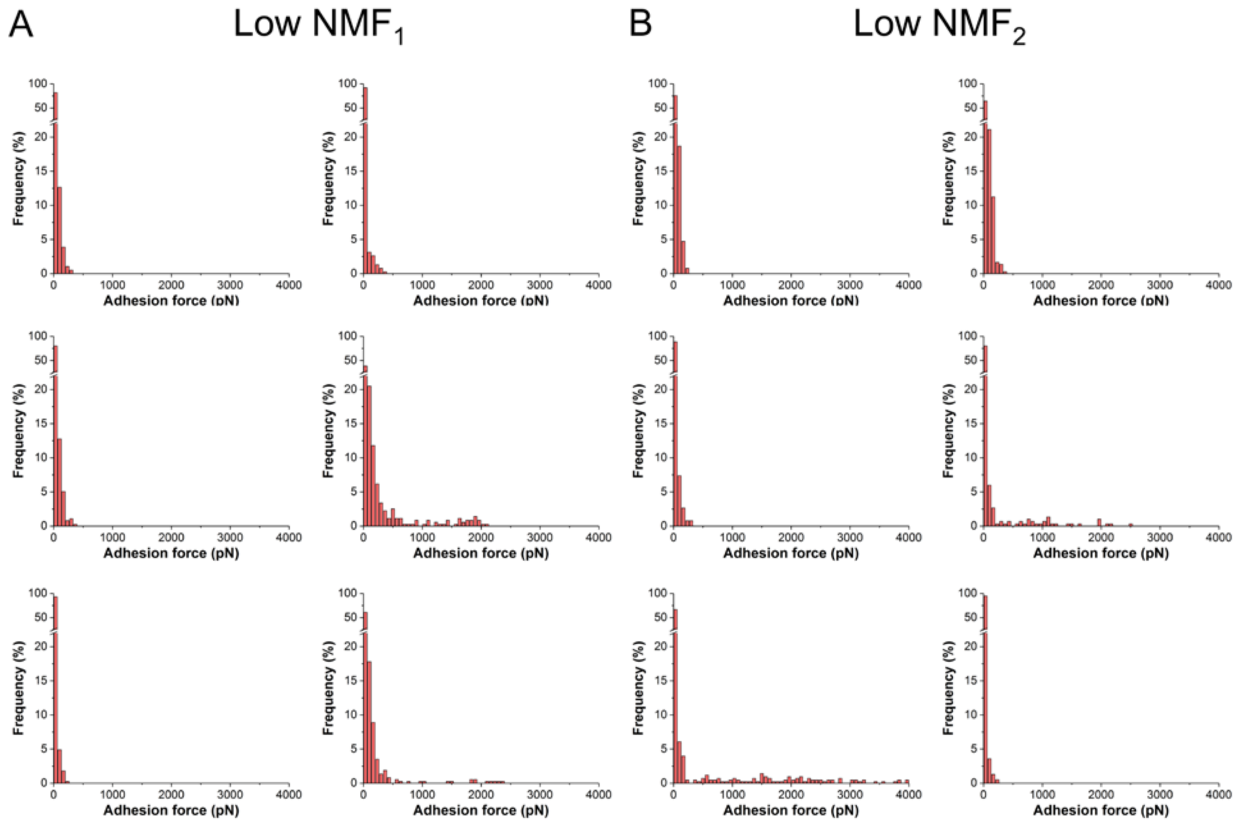

Figure S2. Single-cell force spectroscopy of the interaction between *S. aureus* AD08 Δ*clfB* and AD skins. Adhesion force histograms obtained in PBS between additional *S. aureus* AD08 Δ*clfB* bacteria and corneocytes from AD patients with low NMF levels. For each patient, 6 additional pairs are presented.
