# Supplementary material for: Adhesion of Staphylococcus aureus to Corneocytes from Atopic Dermatitis Patients Is Controlled by Natural Moisturizing Factor Levels
Source: mBio. 2018 Aug 14;9(4):e01184-18. doi: 10.1128/mBio.01184-18 (PMC6094479; doi:10.1128/mBio.01184-18)
Supplement: FIG S3 [file mbo004184009sf3.pdf]

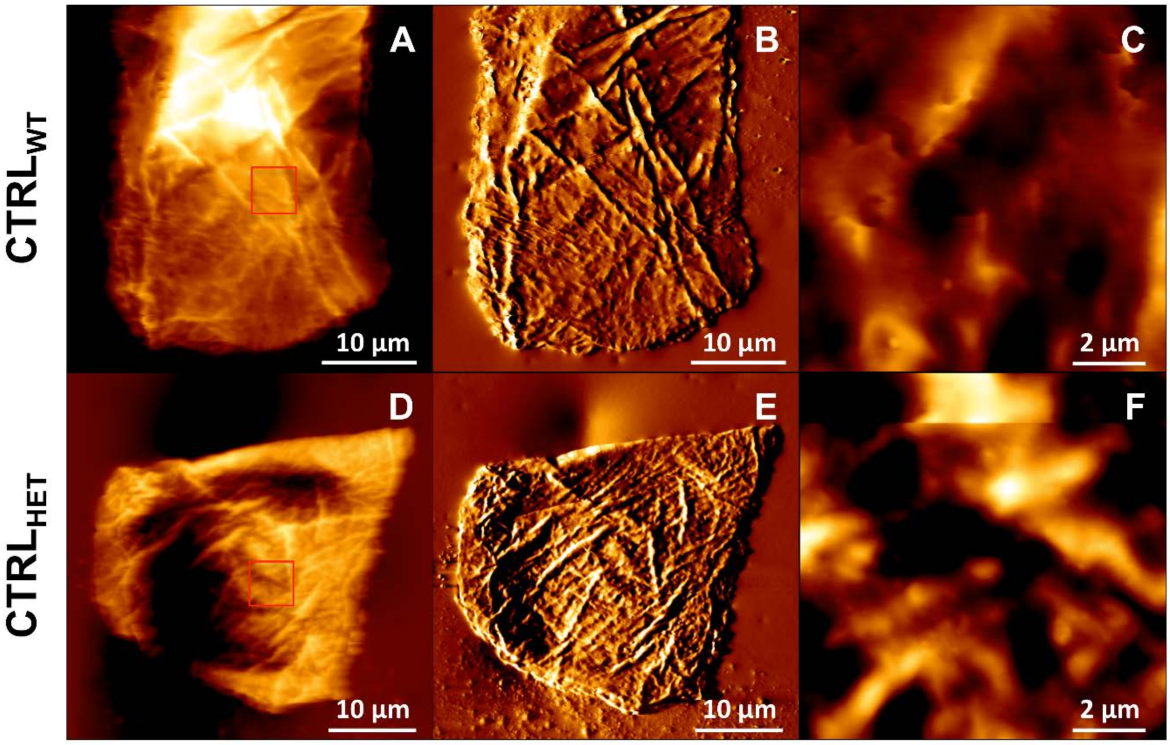

Figure S3. Topographic imaging of non-AD skins. (*A*, *D*) Height images (z-range = 1.5 µm) and (*B*, *E*) corresponding deflection images obtained for corneocytes from children unaffected by AD, and presenting either no detected *FLG* mutation (CTRL$_{WT}$; NMF level = 1.32), or a *FLG* mutation (CTRL$_{HET}$; NMF level = 0.28). (*C*, *F*) High-resolution height images recorded in the square areas shown in (*A*, *D*).
