# Supplementary material for: Adhesion of Staphylococcus aureus to Corneocytes from Atopic Dermatitis Patients Is Controlled by Natural Moisturizing Factor Levels
Source: mBio. 2018 Aug 14;9(4):e01184-18. doi: 10.1128/mBio.01184-18 (PMC6094479; doi:10.1128/mBio.01184-18)
Supplement: FIG S4 [file mbo004184009sf4.pdf]

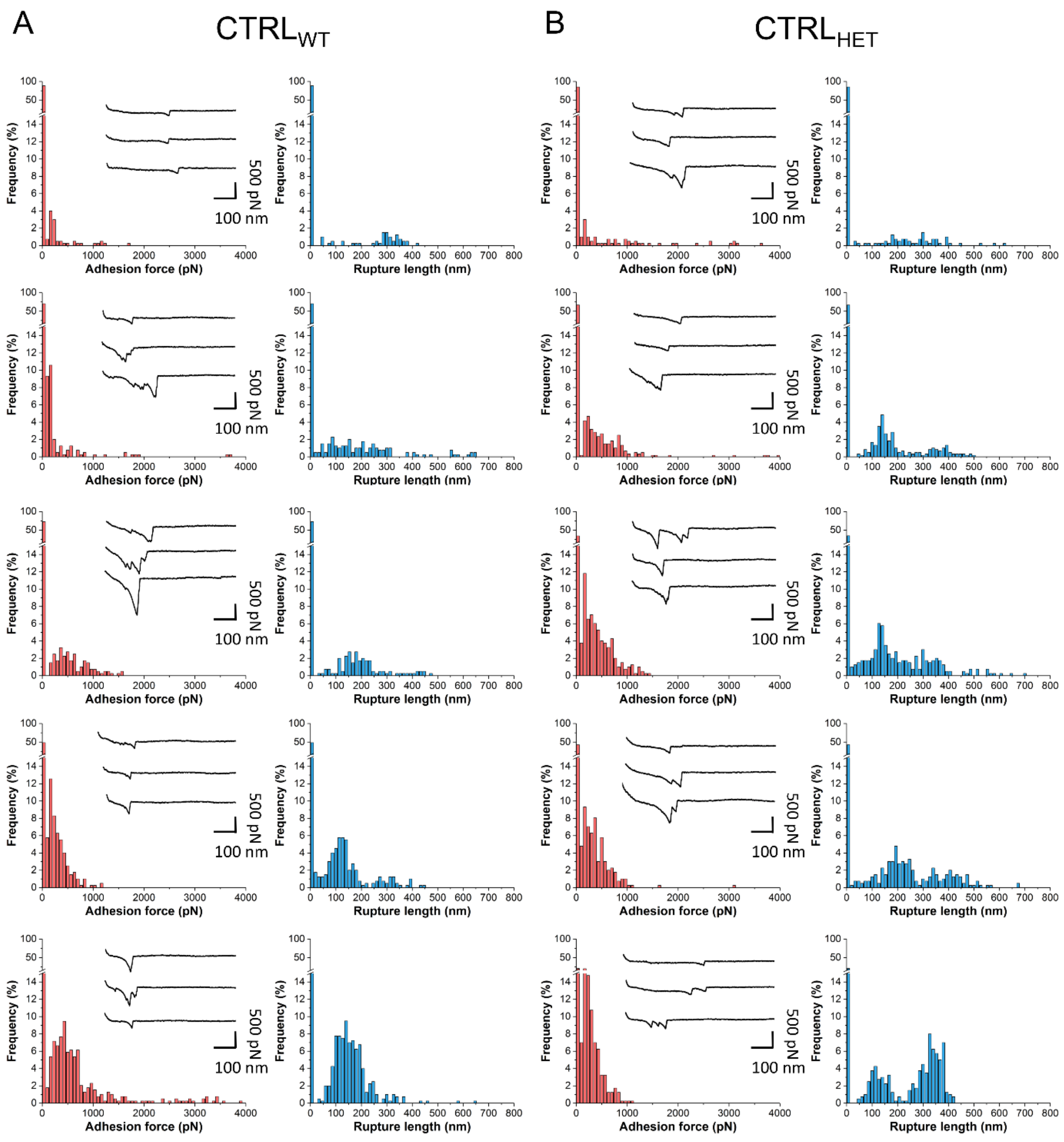

Figure S4. Single-cell force spectroscopy of the interaction between *S. aureus* AD08 and non-AD skins. (*A*, *B*) Adhesion force and rupture distance histograms with representative force profiles obtained in PBS between *S. aureus* AD08 bacteria and corneocytes from children unaffected by AD, CTRL$_{WT}$ (*A*) and CTRL$_{HET}$ (*B*).
