# Supplementary material for: Adhesion of Staphylococcus aureus to Corneocytes from Atopic Dermatitis Patients Is Controlled by Natural Moisturizing Factor Levels
Source: mBio. 2018 Aug 14;9(4):e01184-18. doi: 10.1128/mBio.01184-18 (PMC6094479; doi:10.1128/mBio.01184-18)
Supplement: FIG S5 [file mbo004184009sf5.pdf]

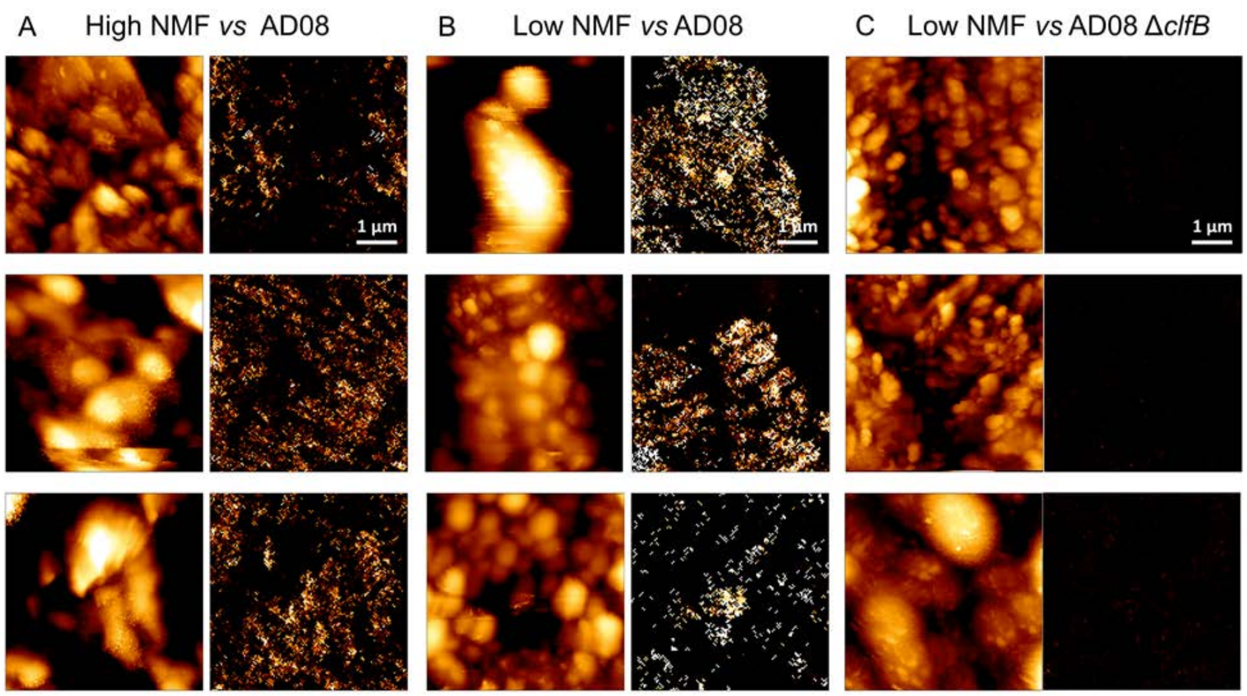

Figure S5. Nanoscale adhesion imaging of AD skins using bacterial probes. (*A*, *B*) Additional height (left) and adhesion (right) images of corneocytes recorded in PBS between *S. aureus* AD08 cell probes and high NMF_2 (*A*) or low NMF_1 (*B*) skin samples. (*C*) Images obtained on low NMF_1 corneocytes with AD08 Δ*clfB* cell probes.
